# Aortic root remodeling and external aortic annuloplasty to treat sinus of Valsalva aneurysm in a patient with complete situs inversus

**DOI:** 10.1186/s13019-015-0352-4

**Published:** 2015-12-07

**Authors:** Charline Pujos, Marie-Catherine Morgant, Ghislain Malapert, Olivier Bouchot

**Affiliations:** Department of Cardiovascular and Thoracic Surgery, Dijon University Hospital, 14 rue Paul Gaffarel, 21079 Dijon cedex, France

**Keywords:** Sinus of Valsalva aneurysm, Congenital heart disease

## Abstract

**Background:**

Sinus of Valsalva aneurysm is an uncommon anomaly of the aorta. It occurs most frequently in the right sinus of Valsalva. Complications depend on its size and location. Situs inversus totalis is a rare condition wherein organs are reversed from their normal positions (mirror image).

**Case presentation:**

We report the case of a 69-year-old man who presented situs inversus totalis known since his childhood and a sinus of Valsalva aneurysm in the right coronary sinus discovered by echocardiography following a history of infection. This was confirmed by CT-scan and MRI. Valve sparing surgery was performed using the remodeling technique associated with external aortic annuloplasty

**Conclusions:**

The remodeling technique with exteranl aortic annuloplasty is usual technique to treat SVA

## Background

Sinus of Valsalva aneurysm (SVA) is the least common of all aortic aneurysms; it is usually a rare congenital anomaly [1]. Surgical repair is recommended due to a possible risk of rupture. Similarly, situs inversus totalis is a rare congenital anomaly. We present herein the case of a patient with known situs inversus totalis associated with right coronary SVA, which was discovered fortuitously.

## Case presentation

The patient was a 69-year-old man with a medical history of repeated urinary tract infections due to the use of a urinary catheter following a transurethral resection of the prostate. The SVA was discovered during a clinical check-up for aortic regurgitation because of suspected infective endocarditis. In addition, this patient had situs inversus totalis known since childhood.

**Fig. 1:**
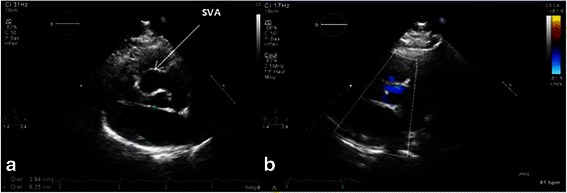
Echocardiography. **a** Showing the Sinus of Valsalva aneurysm (SVA) extending into the right ventricle. **b** Color Doppler showing the aortic regurgitation

The transthoracic echocardiogram (Fig. 1) showed a right SVA of 34 mm prolapsing into the right ventricle, an ascending aorta measuring 43 mm and grade-2 central aortic regurgitation through a tricuspid valve. The left ventricular ejection fraction was preserved.

**Fig. 2:**
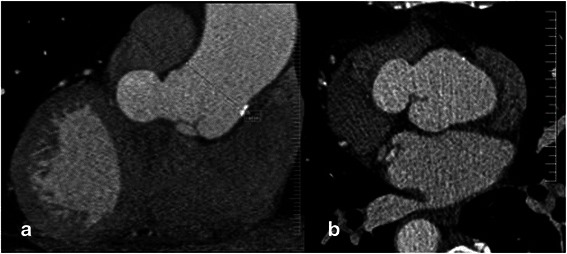
CT scan showing the right coronary sinus aneurysm in the sagittal (**a**) and axial plane (**b**)

**Fig. 3:**
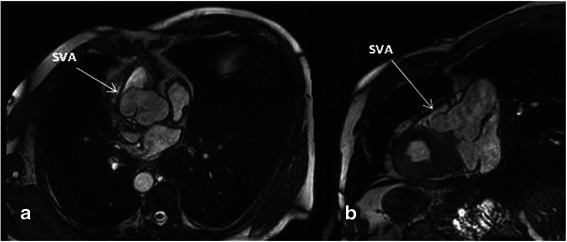
MRI: showing the right coronary sinus aneurysm in the axial (**a**) and sagittal plane (**b**)

Coronary CT angiography (Fig. 2) and Magnetic Resonance Imaging (Fig. 3) showed an aortic root measuring an estimated 50 mm. The ostium of the right coronary artery was located in the anterior left sinus of Valsalva, and the ostium of the left coronary artery was located in the anterior right sinus of Valsalva.

Surgery was indicated because of the association of SVA, moderate dilation of aortic root and ascending aorta, and moderate aortic regurgitation, in a context of chronic urinary infectious disease without proof of aortic valve endocarditis. The procedure was performed through a median sternotomy. Cardiopulmonary bypass was established with intermittent antegrade warm blood myocardial protection.

Peri-operatively, the heart showed complete inversion. The aortic valve was tricuspid, with partial melting at the right and left commissures. The right SVA, which had a pellucid wall, extended into the interventricular septum. It was located right above the insertion of the aortic annulus of the anterior right coronary cusp; the entire aortic valve was dissected and skeletonized with dissection up to the subvalvular plane.

Remodeling of the aortic root with external aortic annuloplasty was performed according to the technique described by Lansac [2]. The distinctive feature in this patient was the inverted appearance of cusps and the absence of aortic tissue in the area of the aneurysm cusp. The Valsalva prosthesis (Gelweave Valsalva, Visintek, UK, 26 mm diameter) was cut to form three scallops. A running suture was performing for each sinus and extending from the nadir up to the commissure. The annuloplasty was done using an Extra Aortic ring (CORONEO®, Montreal, Canada) of 25 mm anchored to the subvalvular plane by five threads. After reimplantation of the coronary arteries, the distal anastomosis was made on the distal ascending aorta. The aortic clamping time was 118 min and the duration of the extracorporeal circulation was 128 min.

The postoperative period was uneventful with no complications. The patient was extubated at 4 H. He was out of the intensive care unit on day 1 and home on day 7. The postoperative echocardiography (day 6 and 1 year) showed no residual aortic regurgitation. After one year, the patient was fine, apart from repeated urinary tract infections due to the use of a urinary catheter. The MRI performed after eleven months showed a good result of the surgical repair.

## Discussion

In a series of patients who underwent cardiac surgery, the incidence of SVA was found to be between 0.15 % and 1.5 % [1]. The etiology may be localized connective tissue dystrophy in the sinuses that causes a rupture between the tunica media and the annulus fibrosus of the aortic valve [3, 4]. This is the case in Marfan’s syndrome (fibrillin) or Ehlers-Danlos syndrome (collagen). In these cases, it is congenital. Secondary causes include atherosclerosis, post-traumatic injuries and medial-cystic necrosis; inflammatory causes such as autoimmune diseases (Horton, Behçet) and infectious causes, including syphilis and infective endocarditis. The aneurysm most often concerns the right coronary sinus (70 %), rarely the non-coronary sinus and, exceptionally, the left coronary sinus (5 %) [3–5]. In 30–60 % of the cases, it is associated with a defect in the interventricular septum and is predominant in men [3–6].

The aneurysmal dilation of the sinuses is gradual and takes place over a number of years. Rupture into the right or left ventricle is the most serious complication of SVA [3, 4]. When they are very large, unruptured aneurysms can obstruct the outflow tract of the right ventricle, or cause myocardial ischemia by compression of the coronary ostium, or a conduction defect, or aortic insufficiency due to distortion of the aortic valve [5, 6].

Situs inversus totalis is a rare congenital anomaly with autosomal recessive transmission wherein organs are reversed from their normal positions. The incidence is estimated at 1/5000 births.

In the literature, only one case of SVA with dextrocardia has been published [7]. It was a ruptured SVA and the patient refused surgery.

Our patient with known situs inversus totalis developed fortuitously discovered right coronary SVA associated with moderate dilation of the aortic root and ascending aorta. The aortic insufficiency, suspected infective endocarditis and the risk of aneurysm rupture led to an indication for surgery.

Direct patch closure could have been considered. Recently, the use of umbrella-like devices has been proposed for treating these aneurysms [8]. However, in this case of SVA combined with aortic regurgitation and moderate dilation of the aortic root and the ascending aorta, we chose remodeling of the aortic root with external aortic annuloplasty, using the technique described by Lansac [2]. In terms of surgical technique, the absence of aortic tissue in the aneurysm area required a suture near the aortic annulus fibrosis, which may be encountered in remodeling for aortic dissection. This surgical technique allows the physiological reconstruction of the aortic root with a good medium-term outcome [2].

## Conclusion

Remodeling of the aortic root associated with external aortic annulopasty to treat SVA associated with dilation of the ascending aorta in this patient with complete situs inversus led to good results.

## Consent

Written informed consent was obtained from the patient for publication of this Case report and any accompanying images. A copy of the written consent is available for review by the Editor-in-Chief of this journal.

## Abbrevations

CT scan: computed tomography
MRI: Magnetic Resonance Imaging
SVA: Sinus of Valsalva aneurysm
